# Tracking Implementation Outcomes of an Intensive Case Management Program for HIV: Protocol for a Mixed Methods Study

**DOI:** 10.2196/57452

**Published:** 2024-11-29

**Authors:** Meron Mengistu, Kris Tom, Liben Gebremikael, Notisha Massaquoi, Obidimma Ezezika

**Affiliations:** 1 Global Health & Innovation Lab Faculty of Health Sciences University of Western Ontario London, ON Canada; 2 TAIBU Community Health Centre Scarborough, ON Canada; 3 Department of Health and Society University of Toronto Scarborough Scarborough, ON Canada

**Keywords:** implementation science, intensive case management, ICM, human immunodeficiency virus, HIV, tracking, outcome, fidelity, reach, sustainability, implementation outcomes, perspective, perception, Toronto, Canada, descriptive statistics, evidence-based intervention, effectiveness, barriers, facilitators, adoption

## Abstract

**Background:**

Implementation science investigates the processes and factors that influence the successful adoption, implementation, and sustainability of interventions in many settings. Although conventional research places significant emphasis on the advancement and effectiveness of interventions, it is equally imperative to comprehend their performance in practical, real-life situations. Through outcome tracking, implementation science enables researchers to investigate complex implementation dynamics and go beyond efficacy, identifying the various aspects that contribute to the success of interventions.

**Objective:**

This study aims to evaluate the implementation outcomes of TAIBU’s intensive case management (ICM) model tailored for African, Caribbean, and Black communities living with HIV in the Greater Toronto Area. Specifically, it seeks to assess the fidelity, reach, and sustainability of the ICM program. Fidelity monitoring will ensure adherence to program protocols and consistency in service delivery, essential for achieving desired health outcomes. Reach assessment will examine the program’s capacity to reach the target population, including demographic coverage and engagement levels among African, Caribbean, and Black individuals. Sustainability assessment will explore the determinants influencing the longevity and impact of the ICM program.

**Methods:**

The study uses a mixed methods approach, where we will use probing questionnaires, interviews, and focus-group discussions to gather program performance and engagement data, in-depth insights, and perspectives from the implementation team responsible for delivering the ICM intervention. The collected fidelity and reach data through questionnaires will be analyzed using appropriate statistical techniques, such as descriptive statistics, to summarize the responses and identify patterns and trends within the data. Sustainability data collected through the interviews and focus groups will be analyzed and organized based on the Consolidated Framework for Implementation Research, which provides an organized way to identify and comprehend the determinants influencing implementation outcomes.

**Results:**

The study commenced in January 2024, and initial data collection is expected to be completed by December 2024. As of September 2024, we have enrolled 5 participants.

**Conclusions:**

This study will significantly contribute to improving the implementation of the ICM program. By conducting a study in an organizational or institutional setting, researchers can acquire valuable insights into the implementation process from those who are directly involved. The information gathered will inform strategies for improving implementation effectiveness; removing impediments; and enhancing the overall quality of the ICM program for African, Caribbean, and Black individuals living with HIV.

**International Registered Report Identifier (IRRID):**

DERR1-10.2196/57452

## Introduction

The 90-90-90 targets were initiated in 2014 by the Joint United Nations Program on HIV and AIDS (UNAIDS) [1]. By 2020, the target was to have 90% of individuals living with HIV diagnosed, provide antiretroviral therapy to 90% of those who were diagnosed, and attain viral suppression for 90% of those who received treatment [1]. The Public Health Agency of Canada suggests that Ontario’s progress in reaching the 90-90-90 targets has been facilitated by increased access to testing, treatment, and health services [2]. However, this overall provincial progress has not translated into similar success among African, Caribbean, and Black communities, which report high HIV prevalence rates, associated with overarching structural factors and barriers to accessing care, predisposing them to new HIV infections [3]. Over the past 10 years, African, Caribbean, and Black peoples have accounted for between 19% and 29% of first-time HIV and AIDS diagnoses in Ontario, while constituting less than 5% of the province’s population [4]. African, Caribbean, and Black populations in Ontario have a higher disease burden, with recent data suggesting that African, Caribbean, and Black people are 12.5% more likely to be infected with HIV than people in the general population, are more likely to experience negative experiences of engagement in care, and are less likely to achieve viral suppression [5]. The uneven and inequitable success of 90-90-90 targets in Ontario suggests that the current evidence-based interventions (EBIs) to improve HIV services may not be culturally or racially effective, may not be translated adequately into practice, or may not be designed to have optimal impact on groups differently impacted by the social determinants of health.

Within the Greater Toronto Area, the TAIBU Community Health Centre has developed and implemented a racially and culturally informed intensive case management (ICM) program for African, Caribbean, and Black communities in the Greater Toronto Area living with HIV [6]. Generally, the ICM model involves promoting independence and quality of life through appropriate services and providing constant ongoing support as needed by the consumer [7]. ICM services can be provided for a wide range of social and health determinants, including mental health, housing challenges, and HIV management [8,9]. Although a standardized definition does not exist, ICM programs share common principles and functions derived from the concept of continuity of care [10]. In the case of TAIBU, the ICM program offers intensive HIV case management, patient navigation and linkage to community supports, access to other support services, peer-led support groups, and HIV education and awareness, along with support to develop personal and coping skills to better meet the unique needs of African, Caribbean, and Black clients [6].

Assessing the outcomes of implementing the TAIBU ICM program is critical to determine its effectiveness, particularly regarding the accessibility and availability of culturally tailored services for African, Caribbean, and Black communities.

To do this, implementation science can bridge the gap between research and practice, ensuring that EBIs are effectively integrated into real-world settings [11]. Central to the success of implementation efforts is the ability to track and evaluate outcomes systematically. Outcome tracking in implementation science enables researchers, practitioners, and policy makers to understand the effectiveness of interventions, identify barriers and facilitators to implementation, and refine strategies to improve the delivery and impact of evidence-based practices [12].

Implementation science outcome tracking is especially crucial for ICM programs. Outcome tracking facilitates the optimization and dissemination of effective ICM interventions by monitoring implementation fidelity, client-level outcomes, influencing factors, and sustainability and by contributing to the evidence base [13]. It equips practitioners, policy makers, and researchers to make informed decisions, enhance program outcomes, and improve the lives of individuals with complex needs. By examining implementation outcomes for African, Caribbean, and Black Canadian individuals with HIV, we can systematically measure key indicators, including accessibility and use of health care services, adherence to antiretroviral therapy, engagement in support programs, viral suppression rates, and overall quality of life [13].

The main goal of this study is to provide support in tracking implementation outcomes in relation to TAIBU’s ICM model, with a specific focus on fidelity, reach, and sustainability.

The overall aim is to gather comprehensive data and insights to inform and improve the implementation of the ICM intervention. The objective is to understand the implementation team’s perspectives, experiences, and perceptions regarding the ICM program and its impact on the target population.

## Methods

### Study Setting and Context

Tracking implementation outcomes within an organization is pivotal for the identification of potential barriers and facilitators to implementation; thus, the setting must be within the organizational or institutional context where the implementation occurs. In this case, the assessment will focus on the TAIBU Community Health Centre, particularly the ICM program that operates out of the center. The ICM program relies on a community-focused approach to HIV care and provides timely and individualized ICM and treatment for people of African, Caribbean, and Black descent who are newly diagnosed with HIV, at risk of HIV, or affected by HIV [6].

The assessment will involve engagement with staff members working in the program, such as the nurse practitioner, nurse, social worker, system navigator, and other relevant stakeholders responsible for implementing and delivering the ICM intervention. Within its context, the study emphasizes understanding the perspectives, experiences, and perceptions of the implementation and delivery team regarding the ICM intervention. The rationale for prioritizing these stakeholders lies in the opportunity to glean valuable insights into the implementation process directly from individuals actively engaged within the organizational or institutional framework. This information can inform strategies for improving implementation effectiveness, addressing barriers, and enhancing the overall quality of the ICM program for African, Caribbean, and Black individuals living with HIV.

### Study Aims

The study included certain specific objectives: (1) identify the barriers, challenges, and facilitators encountered during the implementation process; (2) evaluate the fidelity of the implementation and adherence to established guidelines and protocols (the degree to which the program was implemented as it was prescribed in the original testing or as it was intended by its developers); (3) assess the program’s reach to its intended audience or client base (a measure of the number, proportion, and representativeness of individuals who participated in or were exposed to the program); (4) examine the capacity, resources, and readiness of the implementation team to deliver the ICM intervention effectively; (5) explore the perceptions of the implementation team regarding the sustainability and long-term impact of the ICM program (the program continues to be delivered and individual behavior change is maintained); and (6) gather feedback and suggestions for improving and modifying the ICM intervention and implementation processes.

### Study Design

The study design incorporates both questionnaires and semistructured interviews with the ICM implementation team. The questionnaires will be administered to investigate the fidelity and reach of the ICM program operating out of TAIBU. Furthermore, the study will incorporate semistructured interviews to gather in-depth insights and perspectives from the implementation team about the determinants of program sustainability. Data collected through the interviews (specifically the determinants) will be analyzed and organized based on the Consolidated Framework for Implementation Research (CFIR), which provides a structured approach to identify and understand the factors influencing implementation outcomes.

### Participant Selection and Involvement

The participants in this study are the members of the implementation team responsible for delivering the ICM intervention for African, Caribbean, and Black Canadian individuals with HIV. As participants, they will respond to key questionnaires to share their knowledge, experiences, and perspectives related to the implementation. Their expertise in providing ICM services and their direct involvement in delivering the intervention makes them valuable sources of information.

As the ICM program team is small, we expect our sample size for the study to be small. We anticipate up to 10 participants in the study, given staff time, capacity, and availability. Through their participation, we will gather insights into their understanding of the ICM model, their attitudes toward the intervention, their challenges during implementation, and their perceptions of the barriers and facilitators that influence implementation success. The perspectives and experiences of the implementation team will provide valuable input for understanding the complexities of ICM implementation and informing strategies for improving the intervention’s delivery, overcoming challenges, and ultimately enhancing the quality of care provided to African, Caribbean, and Black Canadian individuals living with HIV.

### Data Collection

#### Overview

In total, 2 separate questionnaires will be administered to capture the fidelity and reach of the ICM program. The participants will complete the questionnaires quarterly, providing their responses to the quantitative criteria established on fidelity and reach. The completed questionnaires will be collected, securely stored, and entered into a Microsoft Excel spreadsheet for analysis. The data will be analyzed using appropriate statistical techniques, such as descriptive statistics, to summarize the responses and identify patterns and trends in the data. Using questionnaires as the data collection tool enables standardized data collection from the implementation team, providing quantitative insights into their perspectives on the implementation of ICM for African, Caribbean, and Black Canadian individuals living with HIV. In addition to the fidelity and reach questionnaires, participants will participate in semistructured interviews to solicit more in-depth and narrative accounts of the program’s determinants associated with sustainability. The semistructured interviews will be conducted once a year, using an interview guide. Each of the 3 measured outcomes (fidelity, reach, and sustainability) will have its own set of questions, with fidelity being the most finely tuned, with an initial probing questionnaire that will eventually develop into a mature and finalized version.

#### Fidelity

Fidelity is a crucial outcome that we will closely monitor, as it ensures consistent program implementation across different settings. It can be defined as the degree to which the program was implemented as it was prescribed in the original testing or as it was intended by its developers [14]. Monitoring fidelity will identify areas that may require improvement and ensure that the program is delivered exactly as intended. Consistency in program delivery is vital to achieving the desired outcomes and minimizing variations that could compromise its effectiveness. For this program, fidelity will be measured using an open-ended questionnaire that relies on the premise of the program conductors understanding the concept of fidelity (Multimedia Appendix 1).

According to Singh and Saldana [15], to achieve the expected health outcomes, it is crucial that providers at EBI delivery sites possess a strong comprehension of the essential program components and the reasons they are needed for. This understanding ensures high-fidelity delivery of these components. Thus, the provided questionnaire will use components based on the criteria for ICM fidelity, resulting in a probing questionnaire intended to evaluate the facilitators’ understanding of fidelity. This questionnaire will be administered only during the initial meeting with the program conductors so as to narrow down the questions that will be administered in later questionnaires. The purpose of the initial questionnaire is to probe the conductors’ understanding of the ICM criteria for fidelity and subsequently create questions that examine that understanding.

#### Reach

The tracking of reach enables us to understand the extent to which the program is reaching its intended audience and identifies disparities in participation rates between different groups. Reach is defined as a measure of the number, proportion, and representativeness of individuals who participated in or were exposed to the program [14]. Such information is invaluable for assessing the program’s impact on the target population and for identifying potential areas for improvement. Because reach is a program outcome that is difficult to evaluate without knowing the extent to which the program is successful in impacting its target population, a questionnaire will be distributed at the end of each quarter to gather quantitative data on the program’s usage and demographics (Multimedia Appendix 2). The form will be sent automatically with the intention of gathering data to test the program’s effectiveness in reaching its intended audience.

#### Sustainability and Determinants

We will also track the determinants of the program’s sustainability, as this outcome is critical for its continuity, resource allocation, maximal impact, stakeholder engagement, and continual learning and improvement. For assessment purposes, sustainability is defined as the program continuing to be delivered and maintained over time [14]. Sustainability will be assessed through semistructured interviews with members of the ICM team, administered annually using an interview guide (Multimedia Appendix 3). The goal is to ensure that the program effectively mitigates the barriers, enhances the facilitators, and addresses the needs of the target population over the long term and makes a lasting positive difference. The Integrated Sustainability Framework will be used in creating the sustainability assessment to devise questions that consider all the relevant domains of ICM [16]. The assessment will be administered only once yearly, as the intention of understanding the determinants of sustainability is relevant only for later purposes when considering scalability. Thus, the 2 primary outcomes that will be tracked throughout the entire course of implementation are reach and fidelity, with the ultimate goal of creating a sustainable program.

#### Implementation Research Logic Model

The Implementation Research Logic Model (IRLM) serves as a helpful tool for assessing program sustainability by organizing determinants that influence sustainability outcomes. By delineating the relationships between determinants, implementation strategies, and mechanisms for implementation within the IRLM framework, researchers can effectively plan and execute strategies to enhance the effectiveness of interventions [17]. This structured approach facilitates a comprehensive understanding of how implementation strategies can be effectively implemented to ultimately improve sustainability outcomes in health care delivery systems. By using the IRLM, the study can effectively identify key determinants, implementation strategies, and mechanisms necessary for enhancing TAIBU’s ICM program outcomes, thus informing specific strategies tailored to improve the overall effectiveness and sustainability of the program.

Successfully populating an IRLM with the corresponding determinants requires taking an initial approach that examines the existing literature and the subsequent expansion of such determinants through data collection (Multimedia Appendix 4). An iterative approach includes (1) reviewing the existing literature, (2) consulting with experts and stakeholders, (3) assessing organizational and contextual factors, (4) analyzing implementation data, (5) engaging with implementation experts and networks, and (6) iteratively refining the model.

Furthermore, the IRLM will be populated using the CFIR, a conceptual framework developed to guide the systematic assessment of multilevel implementation contexts to identify the factors that may influence intervention implementation and effectiveness across five domains: (1) innovation domain, (2) outer setting domain, (3) inner setting domain, (4) individuals’ domain, and (5) implementation process domain.

The innovation domain encompasses determinants related to the innovation being implemented (eg, innovation design and cost). The outer setting domain explores external factors influencing the implementation setting (eg, partnerships and connections, and local conditions). The inner setting domain delineates the implementation setting of the innovation (eg, available resources and mission alignment). The individual’s domain examines the roles and individual characteristics of people involved in or affecting the implementation (eg, opinion leaders and innovation deliverers). Finally, the implementation process domain focuses on the strategies and activities used during implementation (eg, assessing needs and tailoring strategies).

Considering these domains helps to identify potential barriers and facilitators, prioritize areas for intervention, and guide the selection and tailoring of implementation strategies. By considering a wide range of factors, the CFIR supports a comprehensive approach to implementation research and practice, ultimately aiming to enhance the uptake and sustainability of EBIs in real-world settings. This approach will be established through a 4-part questionnaire administered at the beginning of the implementation evaluation to identify the determinants that will fully populate the IRLM. The four-part questionnaire will include the following: (1) What are the barriers to the ICM? (2) What is your opinion on how to overcome the barriers? (3) What are the facilitators? (4) How can these enablers be strengthened?

### Data Analyses

Our data analyses will follow a similar use of qualitative collection tools, including sorting, coding, and interpreting. The full methodology for each of the 3 outcomes is described below.

#### Fidelity

When analyzing fidelity, we follow a systematic approach to organize and interpret the responses obtained from questionnaires. First, we compile all the questionnaire responses and categorize them based on the corresponding questions, providing a clear overview of the data. Next, we read through the responses to identify the recurring themes or patterns that emerge. This involves looking for similarities and differences in the participants’ understanding, implementation experiences, challenges faced, participant engagement strategies, monitoring approaches, and efforts to address the social determinants of health. To further organize and categorize the data, we assign codes or labels to each response that capture the main ideas or themes expressed. This process of coding helps us to summarize the findings effectively. We create a summary or summary table that presents the key findings for each question, highlighting the main themes, notable examples, challenges identified, strategies used, and areas for improvement suggested by the respondents. Finally, we present the results in a clear, concise manner using narrative descriptions and appropriate visual aids, such as charts and graphs. The aim is to ensure that the findings are easily understandable and to highlight the main points of interest. By following this process, we can thoroughly analyze fidelity and provide valuable insights for program implementation.

#### Reach

To assess the reach of the ICM program, several calculations are performed. First, the total enrollment is determined by summing the number of individuals who have enrolled or registered during the reporting period. Second, patient attendance is calculated by adding up the number of patients actively involved in the program in the reporting period. The next step involves identifying return patients by counting the number of individuals who participated in the program more than once during the reporting period. The dropout or discontinuation rate is then determined by counting individuals who discontinued their participation in the program during the reporting period. To gauge the program’s reach, the reach percentage is calculated by dividing the total number of individuals reached by the program during the reporting period by the target population. The result is then multiplied by 100 to obtain the reach percentage. Furthermore, a reach comparison is conducted by comparing the reach achieved in the current reporting period with that of the previous reporting period. Once these calculations have been performed, the data are analyzed and interpreted to obtain insights into the reach of the ICM program. For instance, the enrollment and patient attendance numbers can be compared in order to evaluate the program’s effectiveness in attracting and retaining participants. Similarly, analyzing the dropout or discontinuation rate can identify obstacles or challenges that may impact program engagement. In addition, the reach percentage and reach comparison offer valuable information about the program’s outreach and its growth or decline over time.

#### Determinants and Sustainability

Several steps are taken to assess the sustainability of the ICM program. First, the responses provided by participants are transcribed and organized, ensuring that each response is linked to the corresponding question. These responses are compiled in a document or spreadsheet for analysis. Next, the responses are carefully reviewed to identify common themes and patterns across different questions. We note recurring ideas, perspectives, and observations related to policies, external partnerships, alignment with priorities, program champions, organizational leadership, and organizational infrastructure. To facilitate analysis, the data are then coded, assigning to each response a code or label that captures the main themes or concepts expressed. This process effectively categorizes and organizes the data.

Subsequently, a summary or summary table is created to present the main findings for each question, highlighting the key themes, examples, and insights provided by the participants. Patterns and connections within the responses are analyzed to better understand the identified sustainability factors. We explore relationships between policies, external partnerships, alignment with priorities, program champions, organizational leadership, and organizational infrastructure. The data are further analyzed to derive meaningful insights and interpretations. The implications of the findings for the ICM program’s sustainability are considered by reflecting on the perceived influence of policies, the role of external partnerships and program champions, the support of organizational leadership, and the readiness of the organizational infrastructure for long-term sustainability. The analytical results are then presented in a clear, concise manner, with narrative descriptions or quotes from the participants used as evidence to support the findings. The presentation highlights the main points and provides a comprehensive understanding of the factors influencing the sustainability of the ICM program.

### Ethical Considerations

The Non-Medical Research Ethics Board at the University of Western Ontario has approved this study (121549). Potential study participants will be informed about the study and will provide informed consent before their participation. They will also be informed of their right to withdraw from the study at any time. Questionnaire data, recorded interview files, and observations will be deidentified and securely stored in a designated folder on University of Western Ontario servers inaccessible to unauthorized individuals. Access to the data will be restricted solely to the principal investigator or individuals duly authorized by the principal investigator.

## Results

The study commenced in January 2024, and initial data collection is expected to be completed by December 2024. As of September 2024, we have enrolled 5 participants.

## Discussion

### Expected Findings

The expected outcomes of the assessment encompass gaining deeper insights into TAIBU’s ICM program, especially regarding its implementation, efficacy, and sustainability. These insights will enable the identification of tailored implementation strategies aimed at enhancing the program’s effectiveness in providing care to African, Caribbean, and Black communities within the Greater Toronto Area.

By assessing indicators of fidelity specific to this ICM program, such as caseloads, the use of integrated client-centered support system, and social determinants of health resources, the study seeks to determine whether the program adheres to its prescribed core functions, processes, and policies established during the planning stages. Similarly, examining indicators related to reach, such as levels of active participation and discontinuation rates, offers insights into program use among eligible individuals and the extent to which they maintain engagement. Any discrepancies in fidelity and reach indicators will prompt further investigation into underlying reasons. Through interviews, the study intends to contextualize assessment findings by exploring identified barriers or challenges, aiming to uncover factors influencing low scores in fidelity and reach. Anticipated outcomes of these interviews include identifying determinants and devising strategies to address them, along with mechanisms for implementing these strategies to enhance fidelity and reach outcomes.

The results of this assessment will offer empirical support for the efficacy of ICM programs tailored for African, Caribbean, and Black communities living with HIV—a crucial aspect given the significantly negative HIV-related outcomes experienced within these communities. The results from other studies assessing ICM programs have demonstrated promising outcomes, pointing to the effectiveness of the ICM model in promoting service uptake and engagement, access to social services including employment opportunities and housing, and cost-effectiveness by averting acute inpatient episodes. A systematic review of 40 trials involving 7524 individuals across multiple countries found that ICM interventions tailored to mental health (when compared with standard care) led to improved outcomes, including increased engagement with the service, enhanced general functioning, employment opportunities, reduced homelessness, and shorter hospital stays, especially for those with a history of extended hospitalizations [9]. Furthermore, the findings of an evaluation of ICM programs delivered in Youth Justice Service Centers at Queensland (Australia) identified maintained reductions in reoffending over time and significantly better performance than in alternative youth justice approaches, with approximately 50% greater reduction in reoffending compared with matched cohorts receiving other youth justice supports [18]. ICM has to be proven cost-effective by reducing the frequency and duration of acute inpatient episodes, which can offset its higher service cost when compared with standard services, particularly due to the high expenses associated with acute inpatient treatment [19].

This study demonstrates several strengths, notably its actionability of findings stemming from ongoing monitoring, allowing for the incorporation of emerging strategies throughout the project’s duration. Second, engaging directly with program implementers and deliverers provides valuable firsthand insights into the implementation environment, enriching the understanding of the assessed program’s dynamics. Furthermore, the use of multiple assessment tools, including interviews and questionnaires, ensures a comprehensive examination of the subject matter using both standardized, quantitative measures of outcomes and rich, qualitative narration. However, the study faces several limitations. First, the small sample size may restrict the impact of the interview findings, as the findings emerging may not encompass all possible factors influencing program sustainability. Second, the absence of an observational component limits the depth of understanding, relying solely on participant expression rather than observed actions. In addition, the lack of client satisfaction surveys or direct client engagement limits the ability to contextualize and corroborate the findings, potentially overlooking crucial perspectives from those directly impacted by the program.

Monitoring implementation outcomes generates evidence-based data to inform decision-making and resource allocation, which empowers stakeholders to make informed choices regarding the intervention’s continuation, expansion, or modification based on objective measures of achievement related explicitly to the ICM project. Ultimately, tracking implementation outcomes is crucial to maintaining evidence-based practices; refining the program; and maximizing the intervention’s potential impact on the intended population of African, Caribbean, and Black communities in Toronto.

### Conclusion

This study will contribute significantly to improving the implementation of the ICM program. By conducting the study in an organizational or institutional setting, the researchers will acquire valuable insights into the implementation process from those directly involved. In total, 3 major outcomes will be measured: fidelity, reach, and sustainability. Measuring fidelity will identify areas that may require improvement and ensure that the program is delivered exactly as intended. Measuring reach will identify disparities in participation rates between groups. The information gathered will influence strategies for improving implementation effectiveness; removing impediments; and enhancing the overall quality of the ICM program for African, Caribbean, and Black Canadian individuals living with HIV.

## Appendix

Multimedia Appendix 1 Fidelity questionnaire (responses captured as yes or no).

Multimedia Appendix 2 Reach questionnaire.

Multimedia Appendix 3 Interview guide to understand determinants to sustainability.

Multimedia Appendix 4 The Implementation Research Logic Model for intensive case management.

## Abbreviations

CFIR: Consolidated Framework for Implementation Research
EBI: evidence-based intervention
ICM: intensive case management
IRLM: Implementation Research Logic Model
UNAIDS: Joint United Nations Program on HIV/AIDS

## Data Availability

All data generated or analyzed during this study will be included in the paper as a multimedia appendix.
