# Supplementary material for: Tracking Implementation Outcomes of an Intensive Case Management Program for HIV: Protocol for a Mixed Methods Study
Source: JMIR Res Protoc. 2024 Nov 29;13:e57452. doi: 10.2196/57452 (PMC11645509; doi:10.2196/57452)
Supplement: Multimedia Appendix 1 [file resprot_v13i1e57452_app1.docx]

Multimedia Appendix 1: Fidelity questionnaire (Responses captured as Yes or No)

| **Criteria** | **Indicators** |
| --- | --- |
| Small Caseloads | Case load is less than 20 |
| Initial high- intensity output | Client able to be seen by two service providers (social services provider and healthcare provider) on the same day/visit |
| Integrated Client Centered Support System | Each client (whether at risk, affected by, or living with HIV) has been provided with an individualized and holistic care plan tailored to their specific needs |
|  | For all initial encounters, each service provider uses the screening and client intake templates to collect information from the patient (including med hx, social hc, etc.) |
|  | All clients are referred to additional services if needed through the ICM including primary care, mental health, and support services in addition to HIV care specialist |
| System navigation to  bridge HIV and non-HIV resources | Each client undergoes an assessment by the Systems Navigator within 72hra of intake |
|  | Each client completes an additional assessment during intake by the social worker |
|  | The registered nurse and social worker use the asset map inventory of internal and external non-clinical services as a tool to help with service navigation (e.g. for clients requiring access to food security programs including the Food Bank) |
|  | Each client is provided wrap-around supports required and other potential health and/or social services required in addition to HIV  care (e.g. other health professionals including a dietician, Black social prescriber, chiropractor, food security programs, etc.) |
| Standard set of resources  that is measurable | HIV-specific information for each client whether at risk, affected by, or living with HIV |
|  | Education regarding family planning, medications including PreP, and PEP |
|  | Discussion on maintaining health relationships including sexuality |
| Addressing the impacts of  the Social Determinants  of Health | Each client is able to access their service providers within the ICM team through phone, email, and in-person appointments |
|  | Each client is provided with referrals to individualized programs or partnerships in finding employment and other social services  (based on the social determinant of health being addressed) |
|  | Clients are provided with transportation and food vouchers if they need it |
|  | Family members or partners of clients are actively involved when it's relevant to addressing the client's health and social needs |
