# Supplementary material for: Tracking Implementation Outcomes of an Intensive Case Management Program for HIV: Protocol for a Mixed Methods Study
Source: JMIR Res Protoc. 2024 Nov 29;13:e57452. doi: 10.2196/57452 (PMC11645509; doi:10.2196/57452)
Supplement: Multimedia Appendix 2 [file resprot_v13i1e57452_app2.docx]

Multimedia Appendix 2: Reach questionnaire

| **Question** |
| --- |
| How many individuals were enrolled or registered in the ICM during the reporting period? |
| How many patients actively participated in the program during the reporting period? (e.g., patient attendance) |
| How many return patients were there? |
| How many individuals dropped out or discontinued their participation in the program during the reporting period? |
| What percentage (%) of the target population was reached by the program during the reporting period? |
| How does the reach in the current reporting period compare to the previous reporting period? (Percentage increase or decrease) |
