# Supplementary material for: Tracking Implementation Outcomes of an Intensive Case Management Program for HIV: Protocol for a Mixed Methods Study
Source: JMIR Res Protoc. 2024 Nov 29;13:e57452. doi: 10.2196/57452 (PMC11645509; doi:10.2196/57452)
Supplement: Multimedia Appendix 3 [file resprot_v13i1e57452_app3.docx]

Multimedia Appendix 3: Interview Guide to understand determinants to sustainability

1. What are the barriers to the ICM?
2. What is your opinion on how to overcome the barriers?
3. What are the facilitators?
4. How can these enablers be strengthened?
5. How do you perceive the influence of policies, regulations, and social norms on the sustainability of the ICM Program?
6. Are there any external partnerships (with government agencies, healthcare systems, and community-based organizations) that contribute to the resources, support, and commitment required to sustain the ICM Program? Please provide specific examples.
7. How does the ICM Program align with national, state, and local priorities in terms of sustainability? Are there any ongoing efforts to ensure alignment and support?
8. Describe the role of program champions (community and organizational) in influencing the sustained delivery of the ICM Program. How do they contribute to its sustainability?
9. In your opinion, how does organizational leadership support the long-term sustainability of the ICM Program? Please provide examples or instances that highlight their involvement.
10. Assess the organizational infrastructure (time, financial resources, and space) available to support the ICM Program's sustainability. How prepared is the organization to maintain the program in the long run?
