# Supplementary figures and images for: Tracking Implementation Outcomes of an Intensive Case Management Program for HIV: Protocol for a Mixed Methods Study
Source: JMIR Res Protoc. 2024 Nov 29;13:e57452. doi: 10.2196/57452 (PMC11645509; doi:10.2196/57452)

Multimedia Appendix 4: The Implementation Research Logic Model (IRLM) for ICM



**Fidelity:**

**Reach:**











Supplement: Multimedia Appendix 4 [file resprot_v13i1e57452_app4.docx]
